# Dietary supplementation with silkworm pupae prevents growth retardation in broilers under heat stress conditions

**DOI:** 10.1016/j.psj.2025.106195

**Published:** 2025-12-05

**Authors:** Saita Akanuma, Haruki Nishiguchi, Muhammad Fariz Zahir Ali, Yuki Nagao, Chiemi Miura, Tsutomu Takizawa, Chisato Iida, Akiko Takahashi, Takeshi Miura

**Affiliations:** a Graduate School of Agriculture, Ehime University, 3-5-7, Tarumi, Matsuyama, Ehime, 790-8566, Japan; b Research Center for Marine and Land Bio Industry, National Research and Innovation Agency, Jl. Raya Senggigi, Kodek Bay, Pemenang 83352, Indonesia; c Nissui Corporation, Nishi-Shimbashi Square, 1-3-1, Nishi-Shimbashi, Minato-ku, Tokyo 105-8676, Japan

**Keywords:** Broiler, Heat stress resistance, Insect feed, Bioactive compound

## Abstract

Global warming poses a major challenge to livestock production, particularly in broiler chickens. They are highly sensitive to heat stress due to their elevated metabolic activity associated with rapid growth and high productivity. Pupae of the silkworm (*Bombyx mori*) are known for their immunostimulatory properties. In this study, we investigated the effects on broiler performance of dietary supplementation with defatted silkworm pupae (DSP) under elevated ambient temperatures (neutral, days 0-21; increased temperature, days 21-43). DSP was included in the feed at concentrations of 0.01 % and 0.1 %. Growth performance and mortality were assessed, alongside hepatic transcriptomic analysis using RT-qPCR and RNA sequencing to explore underlying molecular mechanisms. The results showed that broilers fed 0.1 % DSP exhibited an approximately 250 g increase in body weight at 42 days post-hatching, accompanied by increased feed intake (*p* < 0.05). Mortality due to hyperthermia occurred in five broilers in the control group, one in the 0.01 % DSP group, and none in the 0.1 % DSP group. RT-qPCR analysis revealed significantly higher hepatic expression of heat shock proteins (HSPs) in the treatment groups (*p* < 0.05), suggesting improved cellular protection and protein refolding capacity under heat stress. Additionally, pro-inflammatory cytokine expression was downregulated in DSP-fed broilers, while anti-inflammatory cytokine levels were upregulated (*p* < 0.05). RNA sequencing further indicated normalization of hepatic function and induced immune function in the 0.1 % DSP group. These findings suggest that dietary DSP activates the HSP axis by modulating immune responses, contributing to thermotolerance and improved performance in broilers under heat stress.

## Introduction

Broilers are chickens raised specifically for meat production. They are particularly susceptible to high ambient temperatures due to their lack of sweat glands and rapid growth (Mangan and Siwek, 2023). They typically reach body weight suitable for market within six weeks, a process driven by intense energy metabolism and substantial internal heat production, leading to high body temperature (Gous, 2010). Due to its high efficiency of production, the poultry industry plays a critical role in meeting global demand for protein, which is increasing annually due to population growth (Kleyn et al., 2021). However, high ambient temperatures have become a major challenge for poultry farming, resulting in increased mortality, reduced quality of meat, and compromised animal well-being (Goo et al., 2019).

The liver is central for maintaining physiological homeostasis, playing essential roles in energy metabolism, immune responses, and the endocrine system (Desert et al., 2018; Huang et al., 2017). Under heat stress, cells generate reactive oxygen species (ROS) due to the increased ATP synthesis necessary for thermoregulation (Mujahid et al., 2006). Due to its high level of mitochondrial activity, the liver is a major site of injury caused by ROS (Tang et al., 2022). The accumulation of ROS can degrade inhibitors of nuclear factor kappa B (NF-κB), thereby promoting inflammation (Sumint et al., 2009). This can subsequently lead to cell death through the release of damage-associated molecular patterns (DAMPs), further exacerbating inflammatory responses (Rock et al., 2010). Consequently, heat stress-induced hepatic dysfunction increases the risk of reduced growth and increased mortality in broilers.

To mitigate these effects, functional feed additives such as vitamins and polyphenols have been widely investigated for their antioxidant properties, resulting in improvements in growth and immune parameters (Mazur-Kuśnirek et al., 2019). Probiotics, such as *Bacillus* spp. and *Lactobacillus* spp., have been reported to improve growth performance under heat stress by modulating the gut environment (Jahromi et al., 2016; Song et al., 2014) and enhance immune function (Yu et al., 2022). Collectively, these findings highlight a link between immune modulation and heat stress resistance.

In our previous work, we demonstrated the immune-enhancing effects of *Bombyx mori* (silkworm) pupae, leading to the identification of a bioactive compound that activates the innate immune system through NF-κB phosphorylation via Toll-like receptor 4 (TLR4) signaling in RAW264.7 macrophage cell line (Ohta et al., 2016; Ali et al., 2021). Silkworm pupae have been reported as a partial replacement for fish meal as a protein source, with dose-dependent effects on growth promotion in poultry (Banday et al., 2023). In addition, improvements in meat quality (Zsedely et al., 2023) and potential effects on the immune system have also been documented (Zhao et al., 2023). Thus, when the immune modulation is related to heat stress mitigation, silkworm pupae supplementation also has the potential to improve heat stress resistance, such as probiotics supplementation. Other insect species, such as the black soldier fly (*Hermetia illucens*) (Vilela et al., 2021), housefly (*Musca domestica*) (Hong et al., 2020), and mealworm (*Tenebrio molitor*) (Benzertiha et al., 2020), have also shown potential as immunostimulatory feed additives in livestock and aquaculture. However, the role of insect-derived substances mitigating heat stress remains unclear.

In this study, we investigated the effects of dietary supplementation with defatted silkworm pupae (DSP) on broilers reared under neutral to elevated ambient temperatures. Growth performance and hepatic transcriptomic profiles were analyzed to assess the improvement of heat stress resistance and its underlying mechanisms.

## Materials and methods

### Animal rearing and feeding

A total of 198 healthy 0-day-old chicks were purchased from Mori Breeding Farm (Kagawa, Japan) and used in this study. The broilers used in this study were Chunky, which are genetically equivalent to the Ross 308 line. The chicks were randomly divided, with 22 chicks placed in each of nine 1.5-m^2^ cages. Three cages were then assigned to each of the three dietary groups (control, DSP 0.01 %, and DSP 0.1 %), to give a triplicate per group (n = 22 per replicate). After 14 days, the cage size was expanded to 3.0 m^2^. The position of each experimental cage was determined randomly using a random number generator to exclude the effect of environmental factors.

Experimental feeds were formulated based on a commercial experimental diet (SDB No. 1, Feed One, Kanagawa, Japan; Crude protein: 23.8 %, Crude fat: 5.8 %, Crud fiber: 2.5 %, Ash: 5.2 %, Lysine: 1.36 g/100 g, GE: 414 cal/100 g, ME: 307 cal/100 g), which at 21 days post-hatch (dph) was switched to a feed formulated for the later rearing period (SDB No. 2, Feed One, Kanagawa, Japan; Crude protein: 20 %, Crude fat: 6.8 %, Crud fiber: 2.6 %, Crud ash: 5 %, Lysine: 1.09 g/100 g, GE: 413 cal/100 g, ME: 316 cal/100 g). The commercial feed-grade DSP product (Silkrose, Shintoa, Tokyo, Japan) was supplemented to broiler feed depending on the experimental dose. The proximate and amino acid composition of DSP is shown in Table S1. DSP was previously confirmed to exhibit immunostimulatory properties based on nitric oxide (NO) production assays using RAW264.7 cells (Ali et al., 2021).

The room temperature was gradually increased from 25°C to 30°C over 3.5 h on 21 dph, controlled by adjusting the air conditioning. Postmortem examinations of any broilers that died were performed by a veterinarian to identify the causes of death and clinical signs. Death caused by hyperthermia was diagnostically indicated by discolored muscle and filled crop with feed, as results of protein denaturation and sudden death, respectively. The dark-red pulmonary edema observed in the lungs was interpreted as a circulatory disturbance.

Feed intake was measured at 7, 14, 21, 28, 35, and 42 dph. The body weight of all broilers was measured at 7, 21, 28, 35, 42 dph, and the average body weight of broilers was calculated from the total body weight of all birds in each cage at 0 and 14 dph.

### Sampling

All surviving broilers were humanely euthanized by decapitation on 43 dph using appropriate equipment. Hepatic samples were collected and preserved in RNAlater (Thermo Fisher Scientific, AM7021, MA, USA) to inhibit RNase activity and maintain RNA quality for subsequent transcriptome analysis.

### RNA extraction

Liver samples stored in RNAlater were sectioned and homogenized with ISOGENⅡ (Nippon Gene, 311-07361, Tokyo, Japan) using a Precellys 24 bead homogenizer (Bertin Technologies, P002391-P24T0-A.0, Montigny-le-Bretonneux, France). Proteins and impurities were removed according to the ISOGENⅡ protocol using p-Bromoanisole (Fujifilm Wako, 027-16801, Osaka, Japan) and 70 % ethanol precipitation. RNA purity was evaluated using a NanoPhotometer P330 (Implen, P-330-31, Munich, Germany), and acceptable quality was defined by absorbance ratios of A260/A280 and A260/A230 > 1.8. Only high-quality RNA was used for quantitative reverse transcription polymerase chain reaction (RT-qPCR) and RNA sequencing (RNA-seq).

### RT-qPCR

RNA samples were normalized to 500 ng/µL and reverse-transcribed into cDNA using a High-Capacity cDNA Reverse Transcription Kit (Thermo Fisher Scientific, 4374967, MA, USA), according to the manufacturer’s instructions, on a TAdvanced Thermal Cycler (Biometra, 846-x-070-280, Jena, Germany). Primers targeting *heat shock protein 70* (*Hsp70*), *Hsp90, Hsp40*, heat shock factor 2 (*Hsf2*), lipopolysaccharide-induced tumor necrosis factor-alpha factor (*Litaf*), interleukin (IL)-6 (*Il6*), and IL-10 (*Il10*) were designed based on NCBI resources (Table 1). All primers were synthesized by Eurofins Genomics (Tokyo, Japan). Primer amplification efficiency was evaluated, and the optimal dilution for qPCR was determined. RT-qPCR was performed using the CFX96™ Real-Time PCR Detection System (Bio-Rad, 1851196JA, CA, USA) with PowerTrack SYBR Green Master Mix (Thermo Fisher Scientific, A46110, MA, USA). Cycling conditions were 95°C for 2 min (activation), 95°C for 5 sec (denaturation), followed by 40 cycles of 5 sec for annealing and extension. Ct values were determined from amplification curves, and relative gene expression was calculated using the 2^-ΔΔCt^ method, with β-actin as the housekeeping gene.

**Table 1:** Primer sequences used for the quantitative real-time polymerase chain reaction.

Primer name	Primer sequence	Accession number
Hsp70	F: ACAGTGCCCGCTTACTTCAA	AY288299.1
	R: ACACATCAAAAGTGCCCCCT	
Hsp90	F: GAGTTTGACTGACCCGAGCA	NM_001109785.2
	R: TCCCTATGCCGGTATCCACA	
Hsp40	F: TTCACATCCCCAAGTTTAGG	NM_001006685.2
	R: GGCATTCAACAGCATAGA	
Hsf2	F: TGTGGCCTCACTTGCTTCT	NM_001167764.3
	R: CGCTGCTCGCATTCCT	
Litaf	F: GACAGCCTATGCCAACAAGTA	NM_204267.2
	R: GAATTAAGCAACAGCCAGCTATG	
Il6	F: GCGAGAACAGCATGGAGATG	NM_204628.2
	R: GTAGGTCTGAAAGGCGAACAG	
Il10	F: CATGCTGCTGGGCCTGAA	NM_001004414.4
	R: CGTCTCCTTGATCTGCTTGATG	
βactin	F: ACCCCAAAGCCAACAGA	NM_205518.2
	R: CCAGAGTCCATCACAATACC	

### RNA-seq

Genomic DNA was removed using a TURBO DNA-free™ Kit (Thermo Fisher Scientific, AM1907, MA, USA). RNA integrity was confirmed by agarose gel electrophoresis with ethidium bromide, and only samples with an approximate 2:1 ratio of 28S to 18S rRNA were selected for sequencing. RNA-seq was outsourced to Novogene Japan (Tokyo, Japan).

Clean reads were aligned to the reference genome using STAR (ver. 2.7.10b) against the Ensembl database (ver. 1.4). Gene expression was quantified using featureCounts (ver. 3.18). Differentially expressed genes (DEGs) were identified using the TCC package (ver. 1.46.0) with TMM normalization via edgeR in RStudio (ver. 4.4.1). DEGs were defined by |log₂(fold-change) | ≥ 1 and FDR < 0.05. Functional enrichment analysis of DEGs was conducted using clusterProfiler (ver. 4.14.4). Gene Ontology (GO) terms were categorized into biological processes, molecular functions, and cellular components, using the OrgDb annotation. Kyoto Encyclopedia of Genes and Genomes (KEGG) pathway analysis was also performed using clusterProfiler.

### Statistical analysis

Outlier analysis using the Smirnov-Grubbs test was conducted for the RT-qPCR results, as this assay had sufficient replicates (n = 7) to allow the statistically meaningful detection of outliers. One-way ANOVA was used to assess statistical differences in body weight at each time, feed intake, and RT-qPCR data, after testing for homogeneity of variance via Bartlett’s test, using EZR software (Kanda 2012; ver.1.61; https://www.jichi.ac.jp/saitama-sct/SaitamaHP.files/statmed.html, accessed on May 21, 2025). Post-hoc pairwise comparisons were conducted using Bonferroni adjustment to control for multiple testing. Data are presented as means ± SEM (standard error of the mean). Body weight transitions among groups were analyzed using repeated measures data from individual birds at 7, 21, 28, 35, and 42 dph, with a linear mixed model (LMM) implemented in R (R Core Team, 2025; version 4.4.3, https://www.R-project.org/). The model included treatment group, time, and their interaction (group × time) as fixed effects. In addition, the experimental repetition was included as a fixed effect. Bird ID was included as a random intercept to account for subject-level variability.

## Results

### Growth and feed intake

**Fig. 1:**
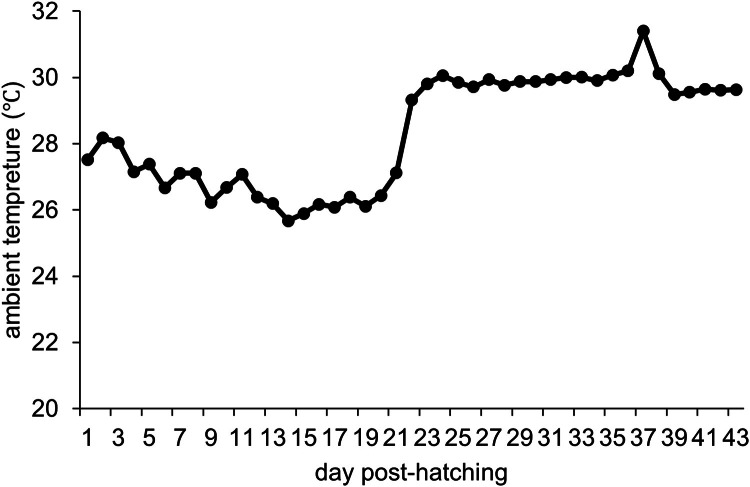
Transition of the daily average ambient temperature, maintained at 30 °C after 21 days post-hatching.

The ambient temperature was maintained at approximately 30°C after 21 dph, except on 37 dph when a temporary equipment malfunction occurred, causing the temperature to rise to a maximum of 34.4°C. (Fig. 1; Fig. S1). Dietary supplementation with 0.1 % DSP resulted in a significantly higher final body weight (*p* < 0.05), approximately 250 g greater than that of the control group (Table 2). DSP supplementation showed significantly higher weight gain than the control group during 21-28 dph (Table 2), which corresponds to the first week after the room temperature was increased at 21 dph.

**Table 2:** Effects of defatted silkworm pupae additive (DSP 0.01 % and 0.1 %), while increasing the temperature to 30°C from 21-day-post-hatching (dph), on body weight, body weight gain, feed intake, and feed conversion rate (FCR). The different letters among feed groups (Control, DSP 0.01 % and 0.1 %, n = 3) in each period indicate significant differences (*p* < 0.05). All data are presented as means ± SEM.

	0 dph	7 dph	14 dph	21 dph	28 dph	35 dph	42 dph
Body weight (g)							
Control	43 ± 0.08	139 ± 2.03	418 ± 6.94	966 ± 15.12	1497 ± 22.19	2119 ± 37.58	2661 ± 45.29^a^
DSP 0.01 %	43 ± 0.12	147 ± 3.92	441 ± 14.08	1011 ± 23.94	1586 ± 30.68	2257 ± 44.74	2837 ± 59.32^ab^
DSP 0.1 %	43 ± 0.31	144 ± 3.06	443 ± 16.12	1017 ± 33.61	1606 ± 36.99	2289 ± 41.4	2908 ± 21.42^b^
body weight gain (g/week/bird)							
Control		95 ± 2.05	280 ± 6.14	548 ± 8.35	531 ± 7.79^a^	621 ± 15.48	542 ± 11.29
DSP 0.01 %		104 ± 4.02	295 ± 10.16	570 ± 9.94	575 ± 10.33^b^	671 ± 18.03	580 ± 16.91
DSP 0.1 %		101 ± 2.91	299 ± 13.54	574 ± 17.68	588 ± 5.23^b^	684 ± 4.56	619 ± 49.52
Feed intake (g/week/bird)							
Control		122 ± 2.98	341 ± 6.96	651 ± 13.08	854 ± 19.04^a^	1038 ± 27.60	1036 ± 23.44^a^
DSP 0.01 %		134 ± 3.98	358 ± 11.55	686 ± 16.83	930 ± 23.08^ab^	1135 ± 28.60	1120 ± 19.11^ab^
DSP 0.1 %		128 ± 4.43	365 ± 15.08	672 ± 14.38	966 ± 23.47^b^	1131 ± 4.10	1172 ± 34.52^b^
Feed conversion rate (Feed intake/Weight gain)							
Control		1.28 ± 0.038	1.22 ± 0.024	1.19 ± 0.009	1.61 ± 0.015	1.67 ± 0.002	1.91 ± 0.017
DSP 0.01 %		1.29 ± 0.036	1.22 ± 0.002	1.20 ± 0.024	1.62 ± 0.024	1.69 ± 0.008	1.93 ± 0.048
DSP 0.1 %		1.27 ± 0.012	1.22 ± 0.005	1.17 ± 0.013	1.64 ± 0.029	1.65 ± 0.030	1.89 ± 0.102

Furthermore, the LMM revealed significant interaction effects between time and DSP treatment (0.01 % and 0.1 %) compared with the control group (*p* < 0.001), indicating an accelerated growth rate throughout the experimental period due to supplementation with DSP (Table 3). In addition, no significant effects of repetition on growth were found in the LMM analysis (p > 0.05) (Table 3).

**Table 3:** Linear mixed model body weight analysis results, including dietary treatment (DSP 0.01 % and 0.1 %), time, and their interaction (treatment × time) as fixed effects, and repetition as an additional fixed effect. Significant interaction terms indicate different growth trajectories in DSP-fed groups compared with the control.

Fixed effect	Estimate	SE	df	t-value	p-value
Intercept	-456.47	31.12	563.64	-14.67	<0.001
DSP 0.01 %	-39.99	43.96	562.29	-0.91	0.363
DSP 0.1 %	-63.91	43.97	562.31	-1.45	0.147
Time	72.63	0.81	758.81	89.74	<0.001
Repetition	7.8961	16.2355	196.2774	0.486	0.627
DSP 0.01 % × time	4.76	1.14	760.11	4.17	<0.001
DSP 0.1 % × time	6.71	1.14	760.57	5.87	<0.001

Abbreviations: df, degrees of freedom; SE, standard error.

Following the increase in ambient temperature, feed intake was significantly higher in the 0.1 % DSP group at 28 and 42 dph (*p* < 0.05) than in the control group, while no differences were observed before the increase in temperature (Table 2). No significant differences in feed conversion ratio (FCR) were observed among the treatment groups throughout the experimental period, although improvements in feed intake and body weight gain were detected (Table 2).

### Mortality

**Fig. 2:**
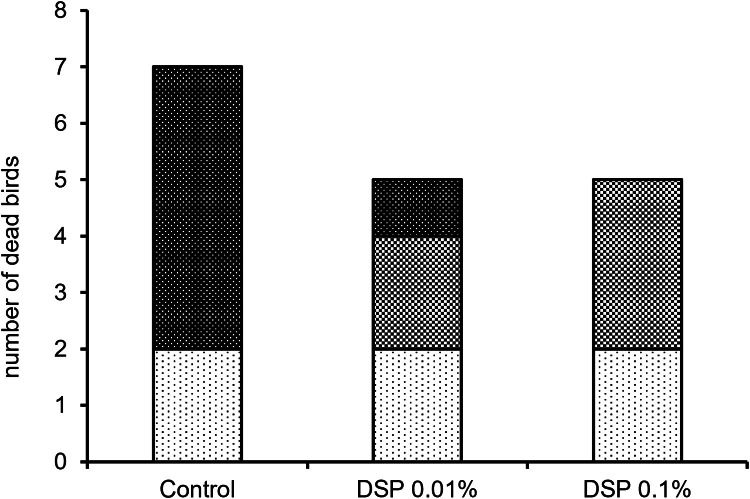
The changing in number of dead birds and results of postmortem examinations dependent on defatted silkworm pupae additive to feed (DSP 0.01 % and 0.1 %). Cause of death: top, hyperthermia; middle, circulatory disturbance; bottom, other reasons.

Over the entire experimental period and repetitions, seven broilers in the control group, five broilers in the 0.01 % DSP group, and five broilers in the 0.1 % DSP group died (Fig. 2). Postmortem examinations revealed that five deaths in the control group and one in the 0.01 % DSP group were due to hyperthermia. Two deaths in the 0.01 % DSP group and three in the 0.1 % DSP group were deemed to be caused by circulatory disturbances, which are known to occur in rapidly growing broilers, especially under high ambient temperatures. In addition, two hyperthermia deaths in the control group also exhibited symptoms of circulatory disturbance.

### Hepatic mRNA expression (RT-qPCR)

**Fig. 3:**
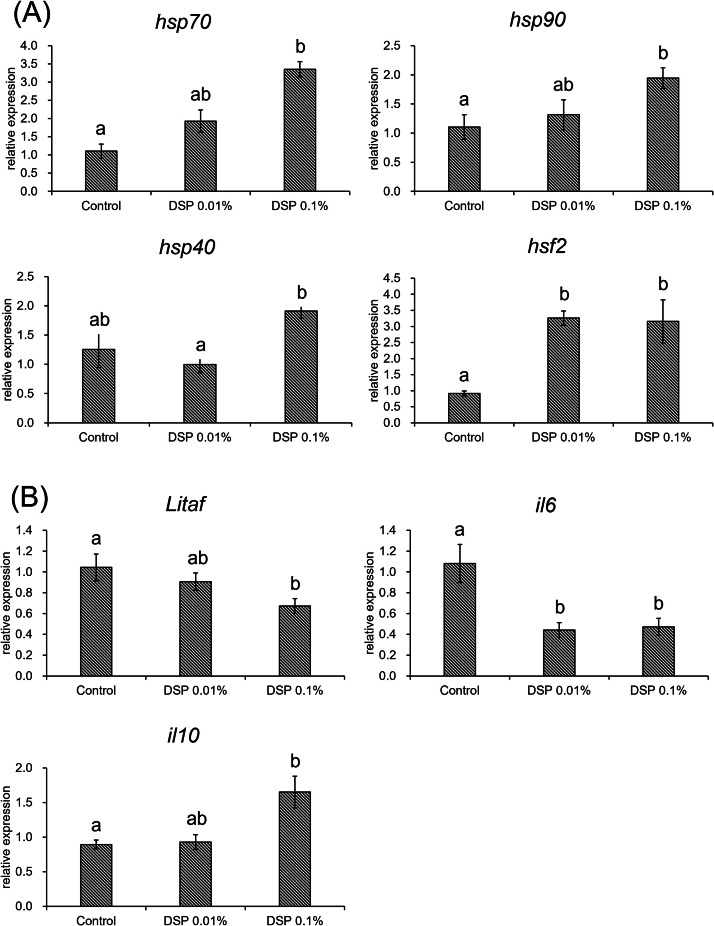
Gene expression in hepatic tissue collected at the end of the experimental period, showing the impact of adding defatted silkworm pupae additive to the feed (DSP 0.01 % and 0.1 %). (A) Relative expression of heat shock proteins (*Hsp70, Hsp90*, and *Hsp40*) and the transcriptome factor (*Hsf2*). (B) Relative expression of pro-inflammatory cytokines (*Il6* and *Litaf*) and an anti-inflammatory cytokine (*Il10*). All data indicate means ± SEM. The different letters among feed groups (Control, DSP 0.01 %, and 0.1 %) indicate significant differences (*p* < 0.05).

To investigate gene expression changes in response to DSP supplementation under high ambient temperature, RT-qPCR and RNA-seq analyses of liver samples collected at 43 dph were conducted. The gene expression of the housekeeping gene didn’t show a difference among groups. DSP supplementation at 0.1 % significantly upregulated the expression of molecular chaperones, including *Hsp70, Hsp90*, and *Hsf2* (*p* < 0.05) (Fig. 3A), although no significant difference was shown on *Hsp40* between the control and the DSP groups. The anti-inflammatory cytokine *Il10* was significantly upregulated in both the 0.01 % and 0.1 % DSP groups (*p* < 0.05) (Fig. 3B). Conversely, the pro-inflammatory cytokines *Litaf* and *Il6* were significantly downregulated in the 0.1 % DSP group compared with the control group (*p* < 0.05) (Fig. 3B).

### RNA-seq and DEG analysis

RNA-seq generated between 44,740,766 and 128,195,444 raw reads per sample. After quality control and adapter trimming, clean reads accounted for 92.17 % ± 0.82 % of the raw reads. The Q20 and Q30 scores were 97.77 % ± 0.48 % and 94.12 % ± 0.62 %, respectively. Clean reads were mapped to the *Gallus gallus* reference genome (GRCg7b), with a mapping rate of 92.17 % ± 0.82 % (Table S2). Differential expression analysis, based on |log₂ fold-change| ≥ 1 and *p* < 0.05, identified 265 upregulated and 600 downregulated genes in the livers of DSP-supplemented broilers compared with those in the control group.

### GO enrichment analysis

GO enrichment analysis was conducted using the clusterProfiler R package. A total of 57 biological processes and 16 molecular functions were significantly enriched. Representative enriched GO terms for biological processes included amino acid metabolic process (GO:0006520), defense response to bacterium (GO:0042742), and NAD⁺ biosynthetic process (GO:0009435); terms for molecular functions included CCR6 chemokine receptor binding (GO:0031731), tetrapyrrole binding (GO:0046906), and vitamin B6 binding (GO:0070279) (Table 4).

**Table 4:** Upregulated Gene Ontology terms related to the metabolism of organic acids, amino acids, nucleotides, and host defenses against pathogens in the 0.1 % defatted silkworm pupae group (DSP 0.1 %).

ID	Description	Gene ratio	q-value
GO:0006082	organic acid metabolic process	21/150	2.51724303886747e-06
GO:0043436	oxoacid metabolic process	20/150	3.32311772129342e-06
GO:0019752	carboxylic acid metabolic process	19/150	8.46662405042317e-06
GO:0006520	amino acid metabolic process	14/150	8.46662405042317e-06
GO:0006753	nucleoside phosphate metabolic process	12/150	0.002748539
GO:0072521	purine-containing compound metabolic process	11/150	0.002137017
GO:0009117	nucleotide metabolic process	10/150	0.002534604
GO:0006790	sulfur compound metabolic process	10/150	0.000633347
GO:0072522	purine-containing compound biosynthetic process	10/150	0.00011935
GO:0009063	amino acid catabolic process	9/150	4.0167642912677e-06
GO:0009069	serine family amino acid metabolic process	5/150	0.000429326
GO:0042742	defense response to bacterium	4/150	0.011957862
GO:0019674	NAD metabolic process	4/150	0.002945102
GO:0072525	pyridine-containing compound biosynthetic process	4/150	0.0014362
GO:0019359	nicotinamide nucleotide biosynthetic process	4/150	0.000633347
GO:0019363	pyridine nucleotide biosynthetic process	4/150	0.000633347
GO:0009070	serine family amino acid biosynthetic process	4/150	0.000544319
GO:0009435	NAD biosynthetic process	4/150	0.000429326
GO:0009064	glutamine family amino acid metabolic process	3/150	0.032958154
GO:0051923	sulfation	3/150	0.012898861
GO:0050830	defense response to Gram-positive bacterium	3/150	0.012898861
GO:0050829	defense response to Gram-negative bacterium	3/150	0.008962692

### KEGG pathway analysis

To explore the effects of DSP supplementation on broader metabolic pathways, KEGG pathway analysis was performed using the same DEGs. The following pathways were significantly enriched: immune system (gga04625), metabolism of cofactors and vitamins (gga00670), and global and overview maps (gga01230).

## Discussion

High ambient temperatures pose a serious threat to poultry, often resulting in reduced feed intake, impaired growth performance, and increased mortality. In the present study, poultry fed with the dietary supplement DSP showed an accelerated growth rate with improved feed intake (Table 2, Table 3). Feed intake is widely considered to be a reliable indicator of physiological stress, which is frequently associated with cellular dysfunction (Maniam & Morris, 2012). Thus, the improved feed intake and survival observed in DSP-fed broilers may indicate that DSP mitigates the stress induced by elevated temperatures. Although no significant differences in FCR were found among the treatment groups (Table 2), a study also reported improvements in feed intake and growth through treatment, without corresponding changes in FCR (Kang et al., 2019). Moreover, a meta-analysis of broiler performance under heat stress reported no consistent correlation between feed intake and FCR (Andretta et al., 2021). Therefore, in our study, while digestibility and energy conversion efficiency may not have been significantly impaired, appetite suppression may have contributed to the reduced growth performance in the control group. However, no groups were reared under neutral ambient temperature, so it is impossible to determine the extent of improvement in the standard growth index, as there is no baseline control. Nevertheless, it is possible to evaluate the effects compared with a heat-stressed control group as a form of mitigation. On the other hand, the necropsy findings from birds that died of hyperthermia confirm that the broilers in this experiment were indeed exposed to heat stress. Therefore, the increased growth and feed intake observed in the DSP-fed group represent outcomes that were obtained under heat stress conditions.

Various feed additives have been studied for their roles in alleviating heat stress, particularly those with antioxidant properties, such as polyphenols and vitamins, which are known to eliminate ROS (Mazur-Kuśnirek et al., 2019). Silkworm pupae, which form the basis of DSP, have been reported to contain polyphenols absorbed from their diet, i.e., mulberry leaves (Aunsha & Negi, 2025; Samappito & Butkhup, 2020). Hence, polyphenol content in 0.1 % DSP is probably not sufficient to match the antioxidant effects observed in studies using purified polyphenol supplementation (Hu et al., 2019), because of the low polyphenol content level in DSP (approximately 3.25 mg/g DSP; Table S1) and low DSP content level in experimental feed. This suggests that polyphenols may not be the primary active compounds responsible for the observed heat stress resistance. The improvement in heat stress tolerance observed in the present study could therefore be attributed to other factors rather than direct antioxidant action from polyphenol.

The RT-qPCR results in the 0.1 % DSP group indicated significantly elevated gene expression of *Hsp70, Hsp90*, and *Hsf2* (Fig. 3A), all of which encode molecular chaperones known to be activated by the accumulation of misfolded proteins. These chaperones play essential roles in preventing protein aggregation and assisting in the refolding of denatured proteins, thereby maintaining cellular homeostasis under stress conditions (Sharp et al., 1999). These genes are considered to be molecular markers of heat shock resistance and have been correlated with enhanced growth performance and reduced cellular damage under thermal stress (Al-Jaryan et al., 2023; Shu et al., 2018). HSP70 is widely known to be genetically conserved, and approximately 88 % of the functional resemblance of HSP70 has been reported between mouse and chicken (Sikiru et al., 2021). These findings suggest that the functional roles of HSP70 reported in mice are largely applicable to chickens. *Hsp70* in particular has been shown to be upregulated in response to inflammatory stimuli, such as lipopolysaccharides (LPS), via the Toll-like receptor pathway and is involved in modulating NF-κB-mediated inflammatory responses through anti-inflammatory signaling cascades in mice (Borges et al., 2012; Dokladny et al., 2010). Consistently, gene expression of *Hsp70* in response to LPS has also been observed in broilers (Wan et al., 2024). A bioactive compound found in DSP is reported to activate immune responses through NF-κB nuclear translocation via the same TLR4 signaling pathway known to be activated by LPS (Ohta et al., 2016). This suggests that the immunostimulant substance in DSP may contribute to the upregulation of *Hsp70*. In addition, Early-stage heat exposure has been reported to enhance subsequent heat stress resistance, which is accompanied by higher expression levels of HSPs compared with broilers that were never exposed to heat (Kang and Shim, 2021). This indicates that stress resistance can be established following prior exposure to stress. In addition, immune stimulation is known to activate pathways similar to those triggered by heat stress because both damage-associated molecular patterns (DAMPs) released under heat stress and pathogen-associated molecular patterns (PAMPs) are recognized by the same pattern-recognition receptors TLR4 (Evans et al., 2015). Therefore, cellular stress resulting from the immune activation induced by the immunostimulatory potential of DSP may contribute to enhanced recovery from subsequent stress caused by high ambient temperature.

Heat stress leads to the generation of ROS and inflammation, often resulting in organ failure (Liu et al., 2022). In addition, compromised intestinal barrier function under thermal stress increases gut permeability, facilitating bacterial translocation into the circulatory system and promoting inflammatory responses in various organs (Nima et al., 2021). Cytokines serve as key mediators in the immune response to infection and cellular damage, with excessive activation, often termed a “cytokine storm”, leading to further ROS generation and tissue damage due to immune cell overactivation (Fajgenbaum & June, 2020). In the current study, RT-qPCR revealed that in DSP-fed broilers, the pro-inflammatory cytokines *Litaf* and *Il6* were downregulated, alongside the upregulation of the anti-inflammatory cytokine *Il10* (Fig. 3B). This expression profile indicates that DSP supplementation modulated the inflammatory response toward homeostasis by suppressing excessive inflammation. In murine studies, *Il10* expression led by HSP70 has been shown to work for anti-inflammatory (Borges et al., 2013), and the anti-inflammatory potential of HSP70 through suppressing nuclear transfer of NF-κB is known (Shi et al., 2006). It may support the proposed Hsp70-related anti-inflammatory mechanism of DSP.

Our transcriptome analysis (RNA-seq) of the broilers’ livers further showed 265 significantly upregulated and 600 significantly downregulated genes. GO enrichment analysis showed that these DEGs were involved in organic acid metabolism, amino acid biosynthesis, nucleotide pathways, NAD⁺ metabolism, and immune responses. Notably, upregulated GO terms were related to amino acid metabolism, such as glutamine family amino acid metabolic process (GO:0006520), serine family amino acid biosynthetic process (GO:0009069), and sulfur compound metabolic process (GO:0006790) (Table 4), which suggests enhancement of the biosynthesis of glutathione, a key antioxidant tripeptide composed of glutamate, cysteine, and glycine. Cysteine and glycine both belong to the serine family. Glutathione is known for its protective role against oxidative stress, particularly under heat stress conditions (Mahmoud & Edens, 2003). Therefore, the enhanced expression of these GO terms suggests that DSP may alleviate heat-induced cellular damage by promoting glutathione synthesis and improving antioxidant capacity. However, the glutathione content was not quantified in this study, which is a limitation. Further confirmation using direct measurements of glutathione levels is needed.

Heat stress is also known to impair mitochondrial function by disrupting the electron transport chain, which leads to increased ROS production due to electron leakage (Akbarian et al., 2016; Huang et al., 2015). This dysfunction results in reduced ATP generation and impaired NAD⁺/NADH cycling (Berger et al., 2004). As a compensatory response, ATP can be synthesized via glycolysis with regenerated NAD⁺, which limits further mitochondrial ROS production (Gohil et al., 2010; Cantó et al., 2015). Under extreme heat stress, animals may also reduce feed intake as a behavioral adaptation to limit metabolic heat and ROS generation (Baumgard & Rhoads, 2013). Therefore, the observed upregulation of GO terms associated with NAD⁺ synthesis in the DSP-fed group (Table 4), combined with a marked reduction in feed intake in the control group, suggests that DSP supplementation may reduce the necessity for metabolic downregulation. By enhancing cellular protection and supporting NAD⁺ cycling, DSP fed to broilers helped them to maintain energy production and growth under heat stress, whereas the control group appeared to rely more on appetite suppression as a defensive strategy.

The immunological effects of silkworm pupae have been reported to enhance serum lysozyme levels in broilers (Lalev et al., 2023) and increase lymphocyte counts in quails (*Coturnix coturnix japonica*) (Anggraeni et al., 2016). Additionally, DSP has been found to contain an immunostimulant that reduces mortality in Japanese medaka (*Oryzias latipes*) following *Edwardsiella tarda* challenge and decreases their intrarenal bacterial load (Ali et al., 2021). In the present study, transcriptomic analysis revealed enrichment of the GO term “defense response to Gram-negative bacterium” (GO:0050830) (Table 4) and the KEGG pathway “C-type lectin receptor signaling” (gga04625), supporting the immunomodulatory role of DSP. The DSP used in this study was confirmed to possess immune-stimulating properties during the manufacturing process. Taken together, these findings suggest that DSP may contribute both to immune enhancement and to cellular protection under heat stress conditions. On the other hand, it should be noted that an unexpected temperature fluctuation occurred at 37 dph, which may have influenced the experimental results.

We primarily focused on gene expression of the liver, whereas protein level dynamics and morphological analyses were not performed. To clarify the contribution of proteins that were shown as regulated genes in this study, further research is required. Additionally, the effects on meat quality, such as drip loss and pH value, which are known to be influenced by heat stress, should also be investigated.

This study demonstrated growth improvement under heat stress by feeding silkworm pupae, which may contribute to reducing housing costs. Additionally, the economic validity of silkworm pupae as a protein source for poultry feed was also reported (Khan, 2018). Adding 0.1 % to feed would not impose a financial burden on farmers because this material is basically a by-product of sericulture.

In conclusion, dietary supplementation with 0.1 % DSP improved growth performance and reduced mortality in broilers under heat stress. Transcriptomic evidence, including increased expression of HSPs and upregulation of energy metabolism-related pathways in the liver, indicated a stress-adaptive response, presumably mediated by mild immunostimulatory activity. Although the precise molecular mechanisms underlying the heat stress-mitigating effects of DSP remain to be fully elucidated, these findings offer a promising strategy for enhancing heat tolerance in poultry, an increasingly relevant goal in the context of global climate change.

## Ethics approval

The rearing of broilers was carried out under a contractual agreement by the Research Institute for Animal Science in Biochemistry and Toxicology (RIAS: https://www.riasbt.jp/en/), in accordance with the Institutional Animal Care and Use Committee (IACUC) guidelines (permit no. 061-000274).

## Data and model availability statement

The raw data are available on the Mendeley Data repository (doi: 10.17632/vt2kkzxp24.1).

## Declaration of generative AI and AI-assisted technologies in the writing process

Generative AI tools (ChatGPT ver.4) were used only for improving the clarity of English expressions.

## Financial support statement

This research was supported by the Nissui Corporation, the Japan Society for the Promotion of Science (JSPS) (grant number 25K02097), and MAFF Commissioned project study on research project for sericultural bio-industry (grant Number JP22680575).

## CRediT authorship contribution statement

**Saita Akanuma:** Writing – original draft, Investigation, Data curation. **Haruki Nishiguchi:** Investigation, Data curation. **Muhammad Fariz Zahir Ali:** Methodology, Formal analysis, Data curation. **Yuki Nagao:** Investigation. **Chiemi Miura:** Project administration, Methodology, Investigation, Formal analysis, Conceptualization. **Tsutomu Takizawa:** Writing – review & editing, Project administration, Methodology, Formal analysis, Conceptualization. **Chisato Iida:** Investigation, Data curation. **Akiko Takahashi:** Investigation, Data curation. **Takeshi Miura:** Writing – review & editing, Supervision, Project administration, Methodology, Investigation, Funding acquisition, Conceptualization.

## Disclosures

The authors declare the following financial interests/personal relationships which may be considered as potential competing interests:

Tsutomu Takizawa, Chisato Iida, Akiko Takahashi reports financial support was provided by Nissui Corporation. Takeshi Miura reports equipment, drugs, or supplies was provided by Nissui Corporation. If there are other authors, they declare that they have no known competing financial interests or personal relationships that could have appeared to influence the work reported in this paper.

## Appendix Supplementary materials

Image, application 1

Image, application 2
