# Supplementary material for: Dietary supplementation with silkworm pupae prevents growth retardation in broilers under heat stress conditions
Source: Poult Sci. 2025 Dec 5;105(1):106195. doi: 10.1016/j.psj.2025.106195 (PMC12752527; doi:10.1016/j.psj.2025.106195)
Supplement: Supplementary file 1 [file mmc1.docx]

Table S1. Proximate composition, amino acid profile, and total polyphenol content of defatted silkworm powder.

| Proximate composition | % |
| --- | --- |
| Moister | 6.2 |
| Crude protein | 73.8 |
| Crude fat | 3.4 |
| Ash | 5.9 |
| Amino acid composition | mg / g Crude protein |
| Arginine | 50 |
| Lysine | 64 |
| Histidine | 29 |
| Phenylalanine | 46 |
| Tyrosine | 58 |
| Leucine | 65 |
| Isoleucine | 39 |
| Methionine | 30 |
| Valine | 50 |
| Alanine | 43 |
| Glycine | 44 |
| Proline | 40 |
| Glutamic acid | 99 |
| Serine | 42 |
| Threonine | 41 |
| Asparatic acid | 96 |
| Tryptophan | 14 |
| Cystein | 14 |
|  | mg / g DSP |
| Total polyphenol | 3.25 |

Table S2. Sequencing quality metrics of RNA-seq data.

|  | Raw reads | clean reads | Q20 | Q30 | uniquely mapped reeds (%) |
| --- | --- | --- | --- | --- | --- |
| DSP-1 | 128195444 | 125298354 | 97.48 | 94.35 | 92.78 |
| DSP-2 | 82779746 | 81022172 | 97.36 | 94.11 | 92.35 |
| DSP-3 | 47927602 | 47493772 | 97.21 | 93.12 | 93.16 |
| control-1 | 45579218 | 45110262 | 98.25 | 94.55 | 91.74 |
| control-2 | 45796586 | 45414656 | 97.98 | 93.73 | 90.83 |
| control-3 | 44740766 | 44223294 | 98.35 | 94.87 | 92.21 |


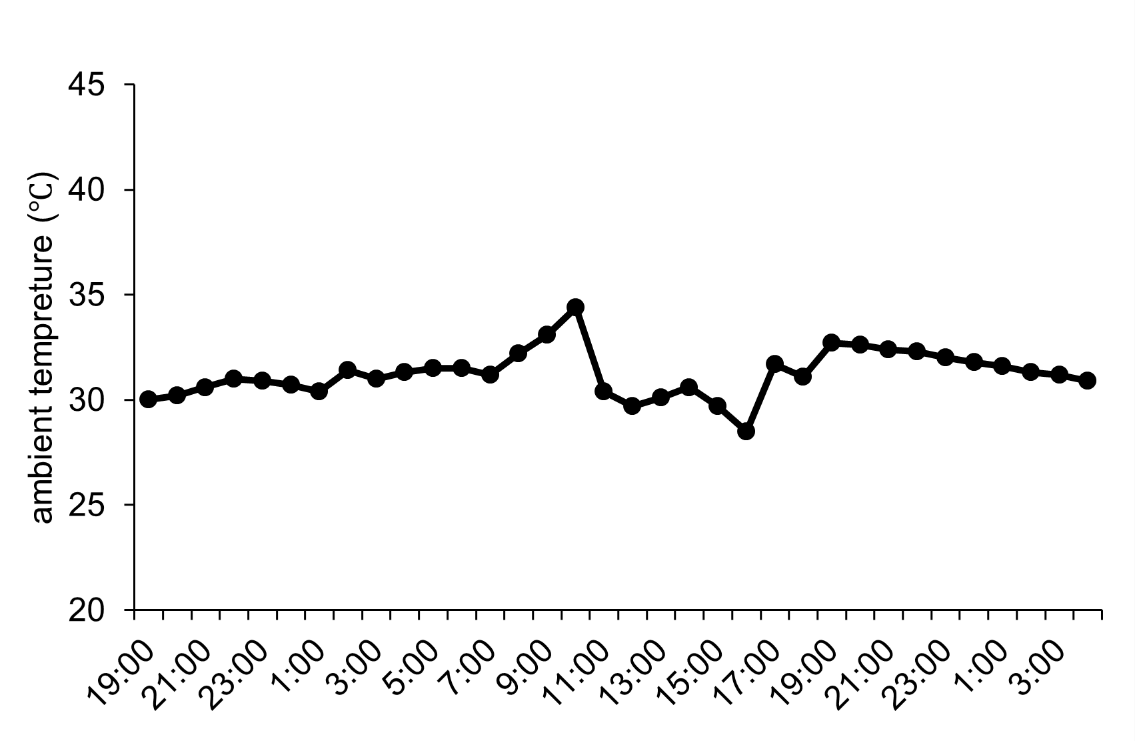


Fig. S1. Ambient temperature fluctuations observed at 37 days post-hatch due to a temporary equipment malfunction.
